# ﻿*Pseudolepraria*, a new leprose genus revealed in Ramalinaceae (Ascomycota, Lecanoromycetes, Lecanorales) to accommodate *Leprariastephaniana*

**DOI:** 10.3897/mycokeys.96.98029

**Published:** 2023-03-24

**Authors:** Martin Kukwa, Magdalena Kosecka, Agnieszka Jabłońska, Adam Flakus, Pamela Rodriguez-Flakus, Beata Guzow-Krzemińska

**Affiliations:** 1 Department of Plant Taxonomy and Nature Conservation, Faculty of Biology, University of Gdańsk, Wita Stwosza 59, PL-80-308 Gdańsk, Poland University of Gdańsk Gdańsk Poland; 2 W. Szafer Institute of Botany, Polish Academy of Sciences, Lubicz 46, PL-31-512 Kraków, Poland W. Szafer Institute of Botany, Polish Academy of Sciences Kraków Poland

**Keywords:** Lichenized fungi, morphology, Neotropics, secondary metabolites, sterile lichens, taxonomy

## Abstract

The new genus *Pseudolepraria* Kukwa, Jabłońska, Kosecka & Guzow-Krzemińska is introduced to accommodate *Leprariastephaniana* Elix, Flakus & Kukwa. Phylogenetic analyses of nucITS, nucLSU, mtSSU and RPB2 markers recovered the new genus in the family Ramalinaceae with strong support. The genus is characterised by its thick, unstratified thallus composed entirely of soredia-like granules, the presence of 4-*O*-methylleprolomin, salazinic acid, zeorin and unknown terpenoid, and its phylogenetic position. The new combination, *P.stephaniana* (Elix, Flakus & Kukwa) Kukwa, Jabłońska, Kosecka & Guzow-Krzemińska, is proposed.

## ﻿Introduction

During the evolution in some groups of lichenized fungi the ability to reproduce sexually has been apparently lost completely and some phylogenetic lineages are known to develop exclusively asexual lichenized propagules. This includes *Lepraria* Ach. (Ascomycota, Lecanoromycetes, Lecanorales, Stereocaulaceae), a well-known genus which up to quite recently comprised only crustose lichens with morphologically simple thalli consisting of soredia-like granules laying directly on substrate or on a layer of hypothalline hyphae (e.g., Ekman and Tønsberg 2002; Kukwa 2002; Sipman 2004; Flakus and Kukwa 2009; Flakus et al. 2011a; Lendemer 2011a, b, 2013a; Lendemer and Hodkinson 2013; Guzow-Krzemińska et al. 2019a). However, Lendemer and Hodkinson (2013) found, based on molecular data, that some fruticose species previously referred to *Leprocaulon* Nyl. also represented *Lepraria* s.str. and they were subsequently transferred to the latter genus. In contrast to their simplified morphology, the species produce a vast variety of secondary lichen metabolites, which are an invaluable tool, together with morphological characters that may be sparse, in the recognition of species and their identification (e.g., Laundon 1989, 1992; Tønsberg 1992; Sipman 2004; Kantvilas and Kukwa 2006; Flakus and Kukwa 2007; Saag et al. 2009; Flakus et al. 2011a; Lendemer 2011a, 2013a; Lendemer and Hodkinson 2013; Guzow-Krzemińska et al. 2019a; Kukwa 2019). It is also noteworthy that some species until recently classified as *Lepraria* have been shown to belong to other genera (e.g., *Leprocaulon* and *Septotrapelia* Aptroot & Chaves; Bungartz et al. 2013; Lendemer and Hodkinson 2013) or even new genera were established for some peculiar species, e.g., *Andreiomyces* Hodkinson & Lendemer within Arthoniomycetes (Hodkinson and Lendemer 2013), *Botryolepraria* Canals et al., related to Verrucariaceae in Eurotiomycetes (Kukwa and Pérez-Ortega 2010) and *Lithocalla* Orange in Lecanorales (probably in Ramalinaceae) in Lecanoromycetes (Orange 2020).

*Lepraria* includes at present c. 75 species (Wijayawardene et al. 2017; Guzow-Krzemińska et al. 2019a; Barcenas-Peña et al. 2021), most of which were described based on chemical (secondary metabolites) and morphological features and some also by molecular markers (e.g., Laundon 1989, 1992; Tønsberg 1992; Lendemer 2011a, 2012, 2013a; Lendemer and Hodkinson 2013; Guzow-Krzemińska et al. 2019a; Barcenas-Peña et al. 2021). One of the species that was placed in *Lepraria* based solely on morphological similarity to other members of the genus was *L.stephaniana* Elix, Flakus & Kukwa (Flakus et al. 2011a). This species is characterised by the thick, unstratified and non-lobed thallus composed of coarse soredia-like granules with soft appearance, and the production of 4-*O*-methylleprolomin, salazinic acid and terpenoids. 4-*O*-methylleprolomin was known only in a single *Pannaria* species before its discovery in *L.stephaniana* (Flakus et al. 2011a). *Leprariastephaniana* has been known until recently only from the type locality, however during field studies in 2017 in Bolivia we found two new localities of the species (one close to the type locality) (Guzow-Krzemińska et al. 2019b). Sequencing of molecular markers of those two recently collected specimens revealed that *L.stephaniana* is unrelated to other species of *Lepraria* s.str., but instead it appeared to be nested within Ramalinaceae as a previously unsequenced lineage close to *Cliostomum* Fr., *Ramalina* Ach. and allied genera. In this paper we introduce the new genus *Pseudolepraria* for this peculiar lineage within Ramalinaceae.

## ﻿Materials and methods

### ﻿Taxon sampling

The studied specimens are deposited in B, BG, KRAM, LPB, NY and UGDA herbaria. Morphology was examined by using Nikon SMZ 800N stereomicroscope. The secondary chemistry of all samples was studied by thin layer chromatography (TLC) following methods by Culberson and Kristinsson (1970) and Orange et al. (2001a).

### ﻿DNA extraction, PCR amplification and DNA sequencing

DNA was extracted using a modified CTAB method (Guzow-Krzemińska and Węgrzyn 2000). We analysed four fungal markers: nucITS rDNA, mtSSU rDNA, nucLSU rDNA, and RPB2 gene. For this purpose we used the following primers: ITS1F (Gardes and Bruns 1993) and ITS4A (Kroken and Taylor 2001) for nucITS rDNA; mrSSU1 and mrSSU3R (Zoller et al. 1999) for mtSSU rDNA; ITS4A-5’ (Kroken and Taylor 2001; Nelsen et al. 2011) and LR5 (Vilgalys and Hester 1990) for nucLSU rDNA; fRPB2-5F and fRPB2-7cR (Liu et al. 1999) for RPB2 gene. Additionally, nucITS rDNA region from green algal partner was amplified using Al1500bf (Helms et al. 2001) and ITS4M primers (Guzow-Krzemińska 2006). PCR was performed in a volume of 25 µl using StartWarm HS-PCR Mix (A&A Biotechnology) following the manufacturer’s protocol. 1 µl of genomic DNA was used for amplification. The PCR cycling parameters are available in Suppl. material 1.

The efficiency of the PCR was checked by visualising the reaction products on a 1% agarose gels stained with SimplySafe (Eurx) dye in order to determine DNA fragment lengths. Afterwards, PCR products were purified using Clean-Up Concentrator (A&A Biotechnology). The sequencing was performed in Macrogen Europe (The Netherlands), using amplification primers. The newly obtained sequences were deposited in GenBank database and their accession numbers are listed in Table 1.

**Table 1.** Species used in this study with their GenBank accession numbers. New sequences are marked in bold.

Species	nucITS rDNA	nucLSU rDNA	mtSSU	RPB2	Algal nucITS rDNA
*Aciculopsorasalmonea*	MG925948	–	MG925842	–	
*Aciculopsorasrilankensis*	MK400258	–	MK400211	–	
*Bacidiaarceutina*	AF282083	MG926041	MG925846	MG926230	
*Bacidiarosella*	AF282086	AY300829	AY300877	AM292755	
*Bacidinaarnoldiana*	AF282093	MG926048	MG925854	MG926238	
*Bacidinaphacodes*	AF282100	MG926049	AY567725	MG926240	
*Badimiadimidiata*	MG925956	MG926052	AY567774	–	
*Bellicidiaincompta*	AF282092	MG926043	MG925849	MG926233	
*Biatoraglobulosa*	AF282073	MG926055	KF662414	KF662450	
*Biatoravacciniicola*	MG925960	MG926060	MG925861	MG926245	
*Biatoravernalis*	AF282070	DQ838752	DQ838753	–	
*Bibbyaalbomarginata*	MG926024	MG926115	MG925927	MG926286	
*Bibbyavermifera*	AF282109	MG926047	MG925852	MG926237	
*Bilimbiasabuletorum*	AM292670	AY756346	AY567721	AM292761	
*Boreoplacaultrafrigida*	HM161512	DQ986797	DQ986813	DQ992421	
*Catillariascotinodes*	AM292673	MG926064	AM292720	AM292763	
*Catinariaatropurpurea*	MG925965	MG926065	MG925865	MG926246	
*Catolechiawahlenbergii*	HQ650649	DQ986794	DQ986811	DQ992424	
*Cenozosiainanis*	–	MG926066	MG925866	–	
*Cliomegalariasymmictoides*	MW622003	MW621867	MW622006	–	
*Cliostomumcorrugatum*	MG925966	MG926067	AY567722	KF662436	
*Cliostomumhaematommatis*	MK446224	–	MK446223	–	
*Eschatogoniaprolifera*	MG925969	MG926070	MG925870	MG926249	
*Kiliasiaathallina*	MG926023	MG926114	–	MG926284	
*Kiliasiasculpturata*	MG926034	MG926122	MG925938	MG926295	
*Krogiacoralloides*	MG925977	MG926072	MG925875	MG926251	
*Lecaniaaipospila*	MG925978	MG926073	MG925876	MG926252	
*Lecaniaerysibe*	AM292682	MG926074	AM292733	AM292769	
*Lecaniafuscella*	AM292684	MG926075	MG925877	–	
*Lecideaalbohyalina*	KF650950	MG926079	KF662398	KF662438	
*Lithocallaecorticata*	KT962179	–	KT962184	–	
*Lithocallamalouina*	KT962178	–	MT857015	–	
*Lueckingiapolyspora*	MG925984	MG926082	MG925882	–	
*Megalariagrossa*	AF282074	MG926083	MG925883	MG926257	
*Megalariaversicolor*	–	AY584651	AY584622	DQ912401	
*Mycobilimbiapilularis*	KF650954	–	KF662402	KF662442	
*Mycobilimbiatetramera*	–	KJ766600	KJ766439	KJ766957	
*Nieblahomalea*	MG925987	–	MG925888	–	
*Namibialinamelanothrix*	MG926038	MG926128	MG925945	MG926303	
*Parallopsorabrakoae*	MG925989	–	MG925891	–	
*Parallopsoraleucophyllina*	MG925994	–	MG925897	MG926265	
*Phyllopsorabreviuscula*	MG925990	MG926087	MG925892	MG926262	
*Phyllopsoragossypina*	MG925967	MG926068	MG925867	MG926247	
*Phyllopsoraparvifoliella*	MG925999	MG926092	MG925902	MG926267	
*Physcidiawrightii*	MN334233	–	MN334227	–	
***Pseudoleprariastephaniana* Kukwa 19740**	**OQ172237**	**OQ172242**	**OQ172251**	–	**OQ303855**
***Pseudoleprariastephaniana* Kukwa 19267**	**OQ172236**	**OQ172243**	**OQ172250**	**OQ160272**	**OQ303854**
*Ramalinadilacerata*	MG926013	MG926104	MG925917	–	
*Ramalinafraxinea*	MG926014	MG926105	MG925918	MG926277	
*Ramalinamannii*	MG926019	MG926111	–	MG926280	
*Ramalinapollinaria*	MG926017	MG926108	AM292752	MG926278	
*Ramalinasinensis*	MG926018	MG926110	MG925921	–	
*Rolfidiumbumammum*	MG926027	MG926117	MG925930	MG926288	
*Ropalosporalugubris*	MG926020	–	MG925922	–	
*Scutulacircumspecta*	–	–	MG925848	–	
*Sporacestrapertexta*	MG926000	MG926093	MG925903	MG926268	
*Stirtoniellakelica*	MG926021	–	MG925923	–	
*Thalloidimacandidum*	AF282117	MG926118	MG925932	MG926290	
*Thalloidimatoninianum*	MG926036	MG926124	MG925942	MG926298	
*Thamnolecaniabrialmontii*	AF282066	MG926112	MG925925	MG926283	
*Toniniacinereovirens*	AF282104	AY756365	AY567724	AM292781	
*Toniniapopulorum*	MG925950	MG926039	MG925843	MG926228	
*Toniniopsisaromatica*	AF282126	MG926113	MG925926	MG926284	
*Toniniopsissubincompta*	AF282125	MG926046	MG925851	MG926236	
*Tylocliostomumviridifarinosum*	NR_174049	–	–	–	
*Tylothalliabiformigera*	AF282077	MG926129	MG925946	MG926304	
*Wayneacalifornica*	–	MG926130	MG925947	MG926305	
*Vermilaciniabreviloba*	MN811352	MN811548	–	MN757330	

### ﻿Sequence alignment and phylogenetic analysis

The newly generated sequences were compared to the sequences available in the GenBank database (http://www.ncbi.nlm.nih.gov/BLAST/) using BLASTn search (Altschul et al. 1990). For the phylogenetic analyses we used representatives of Ramalinaceae and *Boreoplacaultrafrigida* Timdal and *Ropalosporalugubris* (Sommerf.) Poelt were used as outgroup taxa according to previous studies (Kistenich et al. 2018; Orange 2020; van den Boom and Magain 2020). The independent alignments for each marker were generated in MAFFT using auto option and default parameters (Katoh and Standley 2013). The datasets were then subjected to Guidance2 server (Landan and Graur 2008; Penn et al. 2010; Sela et al. 2015; http://guidance.tau.ac.il/) for further analysis. The MSA algorithm was set to MAFFT and 100 bootstrap replicates were used. The Guidance confidence scores were calculated and columns with a score < 0.93 were excluded from the alignments. The terminal ends were trimmed. Single-locus matrices consisted of 61 sequences for nucITS, 62 sequences for mtSSU, 51 sequences for nucLSU, and 44 sequences for RPB2. The best ML tree was inferred for each locus using IQ-TREE with 1000 ultrafast bootstrap replicates as implemented in the IQ-TREE web server (Nguyen et al. 2015; Chernomor et al. 2016; Kalyaanamoorthy et al. 2017; Hoang et al. 2018). Congruence was examined by eye and no significant conflict between loci was observed.

For the final analysis, we concatenated four markers which resulted in a dataset of 66 terminals and 3766 positions. The concatenated dataset was subjected to IQ-TREE analysis to find best-fitting nucleotide substitution models (Nguyen et al. 2015; Chernomor et al. 2016; Kalyaanamoorthy et al. 2017; Hoang et al. 2018). The model selection was restricted to models implemented in MrBayes and the following nucleotide substitution models for the four predefined subsets were selected: GTR+F+I+G for mtSSU rDNA, K2P+I+G for nucITS, and SYM+I+G for both nucLSU rDNA and RPB2 markers. The search for maximum likelihood tree was performed in IQ-TREE and followed with 1000 standard bootstrap replicates (Nguyen et al. 2015; Chernomor et al. 2016; Kalyaanamoorthy et al. 2017; Hoang et al. 2018).

Bayesian analysis was carried out using a Markov Chain Monte Carlo (MCMC) method, in MrBayes v. 3.2.6 (Huelsenbeck and Ronquist 2001; Ronquist and Huelsenbeck 2003) on the CIPRES Web Portal (Miller et al. 2010) using previously selected models (see above). Two parallel MCMC runs were performed, each using four independent chains and ten million generations, sampling every 1000^th^ tree. The resulting log files were analysed using Tracer 1.7.2 (Rambaut et al. 2018). Posterior probabilities (PP) were determined by calculating a majority-rule consensus tree after discarding the initial 25% trees of each chain as the burn-in. The convergence of the chains was confirmed by the convergent diagnostic of the Potential Scale Reduction Factor (PSRF), which approached 1 and the ‘average standard deviation of split frequencies’ was < 0.01).

Phylogenetic trees were visualised using FigTree v. 1.4.3 (Rambaut 2009) and modified in Inkscape (https://inkscape.org/). Bootstrap support (BS values ≥ 70) and PP values (values ≥ 0.95) are given near the branches on the phylogenetic tree. The data were deposited at TreeBASE (Submission ID: 30149).

## ﻿Results and discussion

**Figure 1.**
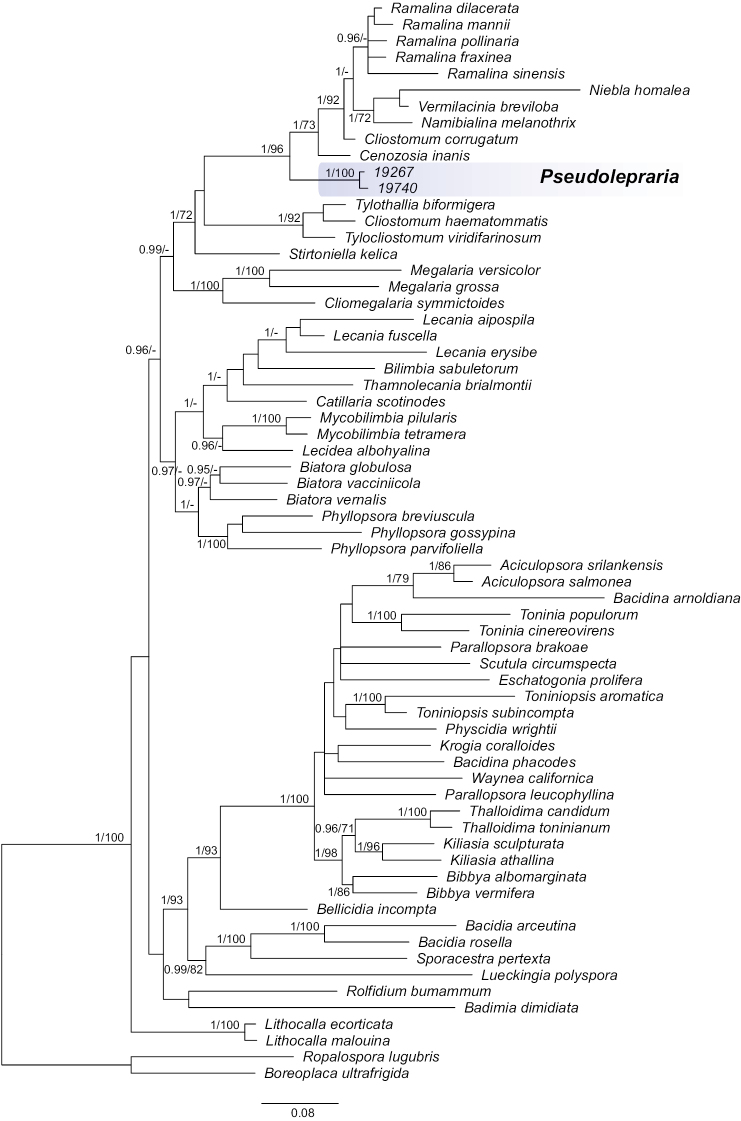
Majority-rule consensus tree resulting from MrBayes analysis of the concatenated mtSSU, nucITS, nucLSU and RPB2 markers with Bayesian PP (values ≥ 0.95) and IQ-TREE bootstrap support values (BS values ≥ 70) given near the branches. *Pseudolepraria* is marked in blue.

For this work we successfully sequenced nucITS, mtSSUand nucLSU from two specimens and additionally RPB2 from one specimen of *Leprariastephaniana* collected in Bolivia (Table 1). BLAST searches of the nucITS, nucLSU, mtSSU and RPB2 markers surprisingly showed the highest similarities to representatives of the family Ramalinaceae, i.e., the genera *Cenozosia* A. Massal., *Cliostomum* and *Ramalina*. Phylogenetic analysis of the concatenated dataset shows that *L.stephaniana* is nested inside Ramalinaceae. The newly sequenced specimens of the species were resolved in a distinct and highly supported clade sister to a clade consisting of *Cliostomum* s.str. represented by the type species *C.corrugatum* (Ach.) Fr., *Cenozosiainanis* (Mont.) A. Massal. and a subclade of several species of *Ramalina*, *Namibialinamelanothrix* (Laurer) Spjut & Sérus., *Niebla* (Ach.) Rundel & Bowler and *Vermilaciniabreviloba* Spjut & Sérus. (Fig. 1). A new monotypic genus, *Pseudolepraria*, is introduced for this lineage of *Leprariastephaniana* and is characterised by a thick, unstratified thallus composed of soredia-like granules, and the presence of 4-*O*-methylleprolomin, salazinic acid, zeorin and unknown terpenoid.

*Pseudolepraria* is the first genus forming leprose and sterile thalli that can be placed with high support within Ramalinaceae. Orange (2020) described the genus *Lithocalla* and placed it with uncertainty in Ramalinaceae. In our phylogeny, *Lithocalla* forms the sister group to Ramalinaceae sensu Kistenich et al. (2018), but this may be an artifact of the taxon sampling. The particular placement of the genus was beyond the scope of this study. *Lithocalla* was introduced for two species, which were originally placed, due to morphological similarities, in *Lepraria*, i.e., *L.ecorticata* (J. R. Laundon) Kukwa and *L.malouina* Øvstedal (Kukwa 2006; Fryday and Øvstedal 2012). Both, *Lithocallaecorticata* (J. R. Laundon) Orange known from Great Britain and Norway, and *L.malouina* (Øvstedal) Fryday & Orange found in the Falkland Islands (Fryday and Øvstedal 2012; Orange 2020), differ from *Pseudoleprariastephaniana*, in their distribution in colder climates, by the production of usnic and fatty acids, the absence of zeorin and the exclusively saxicolous habitat (Orange 2020).

Species resembling *Pseudolepraria* in the Ramalinaceae have up until recently been included in *Crocynia* (Ach.) A. Massal. This genus was established for lichens with a non-corticate, byssoid, felt-like thallus and historically included several species now placed mostly in *Lepraria* (e.g., Laundon 1989, 1992; Kistenich et al. 2018). According to Lücking et al. (2017) *Crocynia* comprised three species and two of them were included in the phylogeny of Ramalinaceae by Kistenich et al. (2018), where they formed a clade nested inside *Phyllopsora* Müll. Arg. Consequently, *Crocynia* was synonymised with *Phyllopsora*, also because of morphological similarities (Kistenich et al. 2018). The status of the third species, *C.microphyllina* Aptroot (Lumbsch et al. 2011), and three species discussed by Sipman (2018) remains uncertain. The species historically placed in *Crocynia* differ from *Pseudolepraria* in the byssoid thalli not producing 4-*O*-methylleprolomin and in sometimes producing apothecia (Cáceres 2007; Lumbsch et al. 2011; Aptroot and Cáceres 2014; Sipman 2018).

*Pseudolepraria* is very similar to *Lepraria* s.str. in sharing the same thallus morphology and, to a certain extent, secondary chemistry (presence of salazinic acid and terpenoids) (e.g., Aptroot 2002; Sipman 2004; Saag et al. 2009; Flakus et al. 2011a; Lendemer 2011a, b). They differ, apart from the phylogenetic position, only in the presence of the very rarely reported 4-*O*-methylleprolomin, a diphenyl ether previously found only in one *Pannaria* species (Flakus et al. 2011a). *Pseudolepraria* differs also in the habitat preferences. It was found only in tropical forests at low elevations (300–470 m a.s.l.), whereas *Lepraria* in tropical South America, including Bolivian ecosystems, are found mostly above 1000 m above sea level (only one locality of *L.finkii* (B. de Lesd.) R.C. Harris found at the elevation of 890 m), in montane forests and open high Andean vegetation (Flakus and Kukwa 2007; Flakus et al. 2011a, b, 2012, 2015; Guzow-Krzemińska et al. 2019a). This is in agreement with the statement presented by Orange et al (2001b), who considered *Lepraria* to be restricted to montane habitats in the tropics.

Poelt (1987) considered the genus *Lepraria* as a ‘box of analogous groups of lichens of completely different origin, held together by the same highly specialized thallus type’. Poelt (1987) also stated that the leprarioid thallus type and the loss of generative reproduction developed in evolution through the reduction of the thallus structures as an adaptation for growing in bark crevices and on over-hanging rocks in ecologically specialised group of lichenized fungi, which includes *Lepraria*, but also, as Poelt (1987) mentioned, *Leproplaca* (Nyl.) Nyl. and some species of the genus *Chrysothrix* Mont. (Poelt 1987). However, this is only partly true, as some lichen groups with this type of thallus (e.g., species of *Leprarianeglecta* group) can grow also in other habitats (e.g., Laundon 1992; Lendemer 2013b). Nevertheless, the statement of Poelt (1987) was true and innovative at this time and it was later shown that the leprarioid thallus indeed originated in several unrelated lichen lineages (e.g., Ekman and Tønsberg 2002; Kukwa and Pérez-Ortega 2010; Hodkinson and Lendemer 2013; Malíček et al. 2018; Guzow-Krzemińska et al. 2017; Orange 2020). Furthermore, some leprarioid genera are known exclusively in sterile state, like *Andreiomyces* (Arthoniales, Arthoniomycetes), *Botryolepraria* (Verrucariales, Eurotiomycetes), *Lepraria* and *Lithocalla* (both in Lecanorales, Lecanoromycetes) (Ekman and Tønsberg 2002; Kukwa and Pérez-Ortega 2010; Hodkinson and Lendemer 2013; Orange 2020). *Pseudolepraria* is another addition to this group, however, as only a few collections are available, it may eventually be found with ascomata.

Buschbom and Mueller (2006) suggested that the asexual way of reproduction is advantageous because the symbiosis with the optimal photobiont for a given environment allows the rapid dissemination of both partners. Therefore, it is more important for the mycobiont to keep the relationship with suitable algal species; however this does not mean that the symbiosis in asexually reproducing species cannot be broken. Kosecka et al. (2021) showed for some *Lepraria* species that the mycobiont can form thalli with different, locally adapted algal strains. We partially sequenced the nucITS region of the photobiont of *Pseudoleprariastephaniana* (Table 1) and found that both thalli associate with the same green algal partner (100% of identity). BLAST hits were closest to *Symbiochloris*, *Dictyochloropsis*, *Massjukichlorella* and *Watanabea* spp., all of which were quite dissimilar to the photobiont sequences of *Pseudoleprariastephaniana*.

## ﻿Taxonomy

### ﻿ Pseudolepraria

Taxon classificationFungiLecanoralesRamalinaceae

Kukwa, Jabłońska, Kosecka & Guzow-Krzemińska gen. nov.

36CF85FA-ED27-57CF-A739-A95AF89D9AF5

847408

#### Diagnosis.

Characterised by thick, unstratified thallus composed of soredia-like granules, the presence of 4-*O*-methylleprolomin, salazinic acid, zeorin, and unknown terpenoid, and the phylogenetic position within Ramalinaceae.

#### Generic type.

*Pseudoleprariastephaniana* (Elix, Flakus & Kukwa) Kukwa, Jabłońska, Kosecka & Guzow-Krzemińska

#### Description.

As this is a monotypic genus the description below constitutes the generic description.

#### Etymology.

The new name refers to the similarity to the genus *Lepraria*, in which this particular species was originally placed.

### ﻿ Pseudolepraria stephaniana

Taxon classificationFungiLecanoralesRamalinaceae

(Elix, Flakus & Kukwa) Kukwa, Jabłońska, Kosecka & Guzow-Krzemińska comb. nov.

E28E76E4-5CFC-51ED-95F9-BFB3CEC406BB

847409

Fig. 2

Lepraria stephaniana Elix, Flakus & Kukwa, in Flakus et al., Lichenologist 43: 64, 2011 (2010). Basionym.

#### Type.

Bolivia. Dept. La Paz: Prov. Iturralde, between Ixiamas and Santa Rosa de Maravillas villages, elev. 305 m, 13°49'16"S, 68°07'18"W, preandean Amazon forest, on bark of tree, 28 July 2008, M. Kukwa 6828 (holotype: UGDA L!; isotypes: B!, BG!, KRAM!, LPB!, NY!).

#### Description

**(adopted from Flakus et al. 2011a).** Thallus crustose, thick, usually not delimited nor lobed, green-grey to creamy-white, not stratified, but sometimes with a poorly differentiated, pseudo-medullary layer of decaying granules. Hypothallus indistinct. Granules coarse with soft appearance, irregularly rounded, up to 100(–200) μm in diam., composed of very lax hyphae mixed with algal cells, usually with projecting hyphae up to c. 30(–50) μm long. Photobiont green, cells globose, up to 12 μm in diam.

#### Chemistry.

Substances detected: 4-*O*-methylleprolomin (major), salazinic acid (minor), zeorin (minor) and an unknown terpenoid (minor) with Rf class values A6, B6, C6. Thallus reactions: K+ yellow turning brownish to red, P+ yellow, C–, KC– (see also Flakus et al. 2011a).

#### Distribution and habitat.

The species is known only from three localities in Bolivia. It was found on bark of trees in transition Chaqueño-Amazon or preandean Amazon forests at elevation between c. 300 to 470 m a.s.l. (Flakus et al. 2011a; Guzow-Krzemińska et al. 2019b).

**Figure 2.**
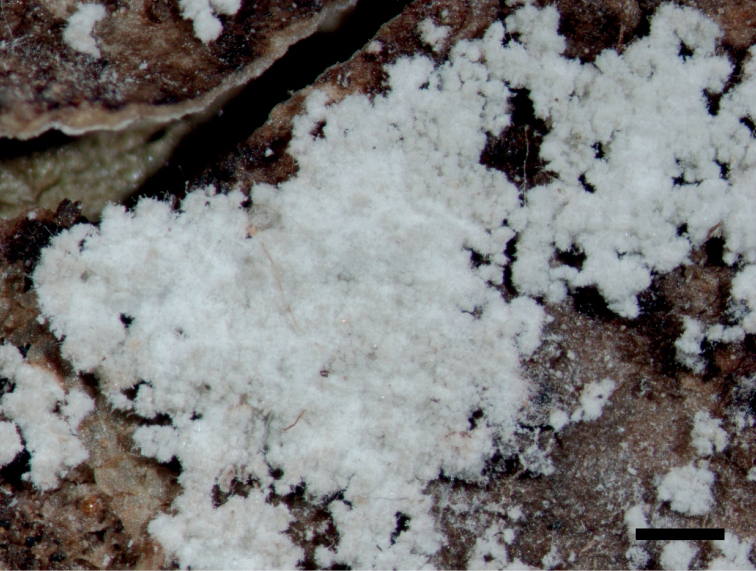
Morphology of *Pseudoleprariastephaniana* (type). Scale bar: 0.5 mm.

#### Specimens used for DNA extraction

**(Table 1).** Bolivia. Dept. La Paz: Prov. Abel Iturralde, between Santa Rosa de Maravillas and Tumupasa, 13°58'43"S, 67°58'14"W, elev. 300 m, natural Preandean Amazon forest, corticolous, 25 May 2017, M. Kukwa 19740 (LPB, UGDA). Dept. Santa Cruz: Prov. Ichilo, Parque Nacional y Área Natural de Manejo Integrado Amboró, Sendero a la Cascada, near Villa Amboró, 17°44'02"S, 63°35'05"W, elev. 470 m, transition Chaqueño-Amazon forest, in the valley, corticolous, 11 May 2017, M. Kukwa 19267 (LPB, UGDA).

## Supplementary Material

XML Treatment for Pseudolepraria

XML Treatment for Pseudolepraria stephaniana

## Supplementary materials

Supplementary material 1 The PCR parameters Data type PCR parameters (word document) Explanation note The PCR parameters are presented for each marker. https://binary.pensoft.net/file/829357This dataset is made available under the Open Database License (http://opendatacommons.org/licenses/odbl/1.0/). The Open Database License (ODbL) is a license agreement intended to allow users to freely share, modify, and use this Dataset while maintaining this same freedom for others, provided that the original source and author(s) are credited.Beata Guzow-Krzemińska, Magdalena Kosecka

## References

[B1] Altschul SF Gish W Miller W Myers EW Lipman DJ (1990) Basic local alignment search tool. Journal of Molecular Biology 215(3): 403–410. 10.1016/S0022-2836(05)80360-2 2231712

[B2] Aptroot A (2002) New and interesting lichens and lichenicolous fungi in Brazil. Fungal Diversity 9: 15–45.

[B3] Aptroot A Cáceres MES (2014) A key to the corticolous microfoliose, foliose and related crustose lichens from Rondônia, Brazil, with the description of four new species. Lichenologist 46(6): 783–799. 10.1017/S0024282914000358

[B4] Barcenas-Peña A Diaz R Grewe F Widhelm T Lumbsch HT (2021) Contributions to the phylogeny of *Lepraria* (Stereocaulaceae) species from the Southern Hemisphere, including three new species. The Bryologist 124(4): 494–505. 10.1639/0007-2745-124.4.494

[B5] Bungartz F Hillmann G Kalb K Elix JA (2013) Leprose and leproid lichens of the Galapagos, with a particular focus on *Lepraria* (Stereocaulaceae) and *Septotrapelia* (Pilocarpaceae). Phytotaxa 150(1): 1–28. 10.11646/phytotaxa.150.1.1

[B6] Buschbom J Mueller GM (2006) Testing “species pair” hypotheses: Evolutionary processes in the lichen-forming species complex *Porpidiaflavocoerulescens* and *Porpidiamelinodes*. Molecular Biology and Evolution 23(3): 574–586. 10.1093/molbev/msj063 16306384

[B7] Cáceres MES (2007) Corticolous crustose and microfoliose lichens of northeastern Brazil. Libri Botanici 22: 1–168.

[B8] Chernomor O von Haeseler A Minh BQ (2016) Terrace aware data structure for phylogenomic inference from supermatrices. Systematic Biology 65(6): 997–1008. 10.1093/sysbio/syw037 27121966 PMC5066062

[B9] Culberson CF Kristinsson H (1970) A standardized method for the identification of lichen products. Journal of Chromatography A 46: 85–93. 10.1016/S0021-9673(00)83967-9 5072880

[B10] Ekman S Tønsberg T (2002) Most species of *Lepraria* and *Leproloma* form a monophyletic group closely related to *Stereocaulon*. Mycological Research 106(11): 1262–1276. 10.1017/S0953756202006718

[B11] Flakus A Kukwa M (2007) New species and records of *Lepraria* (Stereocaulaceae, lichenized Ascomycota) from South America. Lichenologist 39(5): 463–474. 10.1017/S0024282907007116

[B12] Flakus A Kukwa M (2009) *Leprariaglaucosorediata* sp. nov. (Stereocaulaceae, lichenized Ascomycota) and other interesting records of *Lepraria*. Mycotaxon 108(1): 353–364. 10.5248/108.353

[B13] Flakus A Elix JA Rodriguez P Kukwa M (2011a) New species and records of *Lepraria* (Stereocaulaceae, lichenized Ascomycota) from South America. Lichenologist 43(1): 57–66. 10.1017/S0024282910000502

[B14] Flakus A Oset M Jabłońska A Rodriguez Saavedra P Kukwa M (2011b) Contribution to the knowledge of the lichen biota of Bolivia. 3. Polish Botanical Journal 56(2): 159–183.

[B15] Flakus A Etayo J Schiefelbein U Ahti T Jabłońska A Oset M Bach K Rodriguez Saavedra P Kukwa M (2012) Contribution to the knowledge of the lichen biota of Bolivia. 4. Polish Botanical Journal 7(2): 427–461.

[B16] Flakus A Sipman HJM Rodriguez Flakus P Jabłońska A Oset M Meneses QRI Kukwa M (2015) Contribution to the knowledge of the lichen biota of Bolivia. 7. Polish Botanical Journal 60(1): 81–98. 10.1515/pbj-2015-0001

[B17] Fryday AM Øvstedal DO (2012) New species, combinations and records of lichenized fungi from the Falkland Islands (Islas Malvinas). Lichenologist 44(4): 483–500. 10.1017/S0024282912000163

[B18] Gardes M Bruns TD (1993) ITS primers with enhanced specificity for basidiomycetes – application to the identification of mycorrhizae and rusts. Molecular Ecology 2(2): 113–118. 10.1111/j.1365-294X.1993.tb00005.x 8180733

[B19] Guzow-Krzemińska B (2006) Photobiont flexibility in the lichen *Protoparmeliopsismuralis* as revealed by ITS rDNA analyses. Lichenologist 38(5): 469–476. 10.1017/S0024282906005068

[B20] Guzow-Krzemińska B Węgrzyn G (2000) Potential use of restriction analysis of PCR-amplified DNA fragments in taxonomy of lichens. Mycotaxon 76: 305–313.

[B21] Guzow-Krzemińska B Malíček J Tønsberg T Oset M Łubek A Kukwa M (2017) *Lecanorastanislai*, a new, sterile, usnic acid containing lichen species from Eurasia and North America. Phytotaxa 329(3): 201–211. 10.11646/phytotaxa.329.3.1

[B22] Guzow-Krzemińska B Jabłońska A Flakus A Rodriguez-Flakus P Kosecka M Kukwa M (2019a) Phylogenetic placement of *Leprariacryptovouauxii* sp. nov. (Lecanorales, Lecanoromycetes, Ascomycota) with notes on other *Lepraria* species from South America. MycoKeys 53: 1–22. 10.3897/mycokeys.53.33508 31160883 PMC6536479

[B23] Guzow-Krzemińska B Flakus A Kosecka M Jabłońska A Rodriguez-Flakus P Kukwa M (2019b) New species and records of lichens from Bolivia. Phytotaxa 397(4): 257–279. 10.11646/phytotaxa.397.4.1

[B24] Helms G Friedl T Rambold G Mayrhofer H (2001) Identification of photobionts from the lichen family Physciaceae using algal-specific ITS rDNA sequencing. Lichenologist 33(1): 73–86. 10.1006/lich.2000.0298

[B25] Hoang DT Chernomor O Von Haeseler A Minh BQ Vinh LS (2018) UFBoot2: Improving the ultrafast bootstrap approximation. Molecular Biology and Evolution 35(2): 518–522. 10.1093/molbev/msx281 29077904 PMC5850222

[B26] Hodkinson BP Lendemer JC (2013) Next-generation sequencing reveals sterile crustose lichen phylogeny. Mycosphere 4(6): 1028–1039. 10.5943/mycosphere/4/6/1

[B27] Huelsenbeck JP Ronquist F (2001) MRBAYES: Bayesian inference of phylogeny. Bioinformatics 17(8): 754–755. 10.1093/bioinformatics/17.8.754 11524383

[B28] Kalyaanamoorthy S Minh BQ Wong TKF von Haeseler A Jermiin LS (2017) ModelFinder: Fast model selection for accurate phylogenetic estimates. Nature Methods 14(6): 587–589. 10.1038/nmeth.4285 28481363 PMC5453245

[B29] Kantvilas G Kukwa M (2006) A new species of *Lepraria* (lichenized Ascomycetes) from Tasmania’s wet forests. Muelleria 23: 3–6. 10.5962/p.291578

[B30] Katoh K Standley DM (2013) MAFFT: multiple sequence alignment software version 7: improvements in performance and usability. Molecular Biology and Evolution 30(4): 772–780. 10.1093/molbev/mst010 23329690 PMC3603318

[B31] Kistenich S Timdal E Bendiksby M Ekman S (2018) Molecular systematics and character evolution in the lichen family Ramalinaceae (Ascomycota: Lecanorales). Taxon 67(5): 871–904. 10.12705/675.1

[B32] Kosecka M Guzow-Krzemińska B Černajová I Škaloud P Jabłońska A Kukwa M (2021) New lineages of photobionts in Bolivian lichens expand our knowledge on habitat preferences and distribution of *Asterochloris* algae. Scientific Reports 11(1): e8701. 10.1038/s41598-021-88110-0 PMC8062552 33888793

[B33] Kroken S Taylor JW (2001) A gene genealogical approach to recognize phylogenetic species boundaries in the lichenized fungus *Letharia*. Mycologia 93(1): 38–53. 10.1080/00275514.2001.12061278

[B34] Kukwa M (2002) Taxonomic notes on the lichen genera *Lepraria* and *Leproloma*. Annales Botanici Fennici 39: 225–226.

[B35] Kukwa M (2006) Notes on taxonomy and distribution of the lichen species *Leprariaecorticata* comb. nov. Mycotaxon 97: 63–66.

[B36] Kukwa M (2019) *Leprariajuanfernandezii*, a new lichen species from the Southern Hemisphere. Plant and Fungal Systematics 64(2): 233–235. 10.2478/pfs-2019-0019

[B37] Kukwa M Pérez-Ortega S (2010) A second species of *Botryolepraria* from the Neotropics and the phylogenetic placement of the genus within Ascomycota. Mycological Progress 9(3): 345–351. 10.1007/s11557-009-0642-0

[B38] Landan G Graur D (2008) Local reliability measures from sets of co-optimal multiple sequence alignments. Pacific Symposium on Biocomputing 13: 15–24. 18229673

[B39] Laundon JR (1989) The species of *Leproloma*-the name for the *Leprariamembranacea* group. Lichenologist 21(1): 1–22. 10.1017/S0024282989000034

[B40] Laundon JR (1992) *Lepraria* in the British Isles. Lichenologist 24(4): 315–350. 10.1017/S002428299200046X

[B41] Lendemer JC (2011a) A taxonomic revision of the North American species of *Lepraria* s.l. that produce divaricatic acid, with notes on the type species of the genus *L.incana*. Mycologia 103(6): 1216–1229. 10.3852/11-032 21642343

[B42] Lendemer JC (2011b) A standardized morphological terminology and descriptive scheme for *Lepraria* (Stereocaulaceae). Lichenologist 43(5): 379–399. 10.1017/S0024282911000326

[B43] Lendemer JC (2012) Perspectives on chemotaxonomy: Molecular data confirm the existence of two morphologically distinct species within a chemically defined *Leprariacaesiella* (Stereocaulaceae). Castanea 77(1): 89–105. 10.2179/11-042

[B44] Lendemer JC (2013a) A monograph of the crustose members of the genus *Lepraria* Ach. s. str. (Stereocaulaceae, Lichenized Ascomycetes) in North America north of Mexico. Opuscula Philolichenum 12(1): 27–141.

[B45] Lendemer JC (2013b) Shifting paradigms in the taxonomy of lichenized fungi: Molecular phylogenetic evidence corroborates morphology but not chemistry in the *Leprarianeglecta* group. Memoirs of the New York Botanical Garden 108: 127–153.

[B46] Lendemer JC Hodkinson BP (2013) A radical shift in the taxonomy of *Lepraria* s.l.: Molecular and morphological studies shed new light on the evolution of asexuality and lichen growth form diversification. Mycologia 105(4): 994–1018. 10.3852/12-338 23709574

[B47] Liu YJ Whelen S Hall BD (1999) Phylogenetic relationships among ascomycetes: Evidence from an RNA Polymerase II Subunit. Molecular Biology and Evolution 16(12): 1799–1808. 10.1093/oxfordjournals.molbev.a026092 10605121

[B48] Lücking R Hodkinson BP Leavitt SD (2017 [2016]) The 2016 classification of lichenized fungi in the Ascomycota and Basidiomycota – Approaching one thousand genera. The Bryologist 119(4): 361–416. 10.1639/0007-2745-119.4.361

[B49] Lumbsch HT Ahti T Altermann S Amo De Paz G Aptroot A Arup U Bárcenas Peña A Bawingan PA Benatti MN Betancourt L Björk CR Boonpragob K Brand M Bungartz F Cáceres MES Candan M Chaves JL Clerc P Common R Coppins BJ Crespo A Dal-Forno M Divakar PK Duya MV Elix JA Elvebakk A Fankhauser JD Farkas E Itatí Ferraro L Fischer E Galloway DJ Gaya E Giralt M Goward T Grube M Hafellner J Hernández M JE Herrera Campos MA Kalb K Kärnefelt I Kantvilas G Killmann D Kirika P Knudsen K Komposch H Kondratyuk S Lawrey JD Mangold A Marcelli MP McCune B Messuti MI Michlig A Miranda González R Moncada B Naikatini A Nelsen MP Øvstedal DO Palice Z Papong K Parnmen S Pérez-Ortega S Printzen C Rico VJ Rivas Plata E Robayo J Rosabal D Ruprecht U Salazar Allen N Sancho L Santos De Jesus L Santos Vieira T Schultz M Seaward MRD Sérusiaux E Schmitt I Sipman HJM Sohrabi M Søchting U Søgaard MZ Sparrius LB Spielmann A Spribille T Sutjaritturakan J Thammathaworn A Thell A Thor G Thüs H Timdal E Truong C Türk R Umaña Tenorio L Upreti DK van den Boom P Vivas Rebuelta M Wedin M Will-Wolf S Wirth V Wirtz N Yahr R Yeshitela K Ziemmeck F Wheeler T Lücking R (2011) One hundred new species of lichenized fungi: a signature of undiscovered global diversity. Phytotaxa 18: 1–127. 10.11646/phytotaxa.18.1.1

[B50] Malíček J Palice Z Vondrák J Łubek A Kukwa M (2018) *Bacidiaalbogranulosa* (Ramalinaceae, lichenized Ascomycota), a new sorediate lichen from European old-growth forests. MycoKeys 44: 51–62. 10.3897/mycokeys.44.30199 PMC6303282 30595657

[B51] Miller MA Pfeiffer W Schwartz T (2010) Creating the CIPRES Science Gateway for inference of large phylogenetic trees. 2010 Gateway Computing Environments Workshop (GCE). 14 Nov. 2010. New Orleans Convention Center, New Orleans, 8 pp. 10.1109/GCE.2010.5676129

[B52] Nelsen MP Lücking R Mbatchou JS Andrew CJ Spielmann AA Lumbsch TH (2011) New insights into relationships of lichen-forming Dothideomycetes. Fungal Diversity 51(1): 155–162. 10.1007/s13225-011-0144-7

[B53] Nguyen LT Schmidt HA Von Haeseler A Minh BQ (2015) IQ-TREE: A fast and effective stochastic algorithm for estimating maximum-likelihood phylogenies. Molecular Biology and Evolution 32(1): 268–274. 10.1093/molbev/msu300 25371430 PMC4271533

[B54] Orange A (2020) *Lithocalla* (Ascomycota, Lecanorales), a new genus of leprose lichens containing usnic acid. Lichenologist 52(6): 425–435. 10.1017/S0024282920000419

[B55] Orange A James PW White FJ (2001a) Microchemical Methods for the Identification of Lichens. British Lichen Society, London, 101 pp.

[B56] Orange A Wolseley P Karunaratne V Bombuwala K (2001b) Two leprarioid lichens new to Sri Lanka. Bibliotheca Lichenologica 78: 327–333.

[B57] Penn O Privman E Ashkenazy H Landan G Graur D Pupko T (2010) GUIDANCE: a web server for assessing alignment confidence scores. Nucleic Acids Research 38: W23–W28. 10.1093/nar/gkq443 PMC2896199 20497997

[B58] Poelt J (1987) On the reduction of morphological structures in lichens. Bibliotheca Lichenologica 25: 35–45.

[B59] Rambaut A (2009) FigTree ver. 1.4.3. http://tree.bio.ed.ac.uk/software/figtree [Accessed 4 Oct. 2016]

[B60] Rambaut A Drummond AJ Xie D Baele G Suchard MA (2018) Posterior summarisation in Bayesian phylogenetics using Tracer 1.7. Systematic Biology 67(5): 901–904. 10.1093/sysbio/syy032 29718447 PMC6101584

[B61] Ronquist F Huelsenbeck JP (2003) MrBayes 3: Bayesian phylogenetic inference under mixed models. Bioinformatics 19(12): 1572–1574. 10.1093/bioinformatics/btg180 12912839

[B62] Saag L Saag A Randlane T (2009) World survey of the genus *Lepraria* (Stereocaulaceae, lichenized Ascomycota). Lichenologist 41(1): 25–60. 10.1017/S0024282909007993

[B63] Sela I Ashkenazy H Katoh K Pupko T (2015) GUIDANCE2: accurate detection of unreliable alignment regions accounting for the uncertainty of multiple parameters. Nucleic Acids Research 43: W7–W14. 10.1093/nar/gkv318 PMC4489236 25883146

[B64] Sipman HJM (2004) Survey of *Lepraria* species with lobed thallus margins in the tropics. Herzogia 17: 23–35.

[B65] Sipman HJM (2018) Three new lichen species and 48 new records from Vanuatu. Australasian Lichenology 82: 106–129.

[B66] Tønsberg T (1992) The sorediate and isidiate, corticolous, crustose lichens in Norway. Sommerfeltia 14(1): 1–331. 10.2478/som-1992-0002

[B67] van den Boom P Magain N (2020) Three new lichen species from Macaronesia belonging in Ramalinaceae, with the description of a new genus. Plant and Fungal Systematics 65(1): 167–175. 10.35535/pfsyst-2020-0011

[B68] Vilgalys R Hester M (1990) Rapid genetic identification and mapping of enzymatically amplified ribosomal DNA from several *Cryptococcus* species. Journal of Bacteriology 172(8): 4238–4246. 10.1128/jb.172.8.4238-4246.1990 2376561 PMC213247

[B69] Wijayawardene NN Hyde KD Rajeshkumar KC Hawksworth DL Madrid H Kirk PM Braun U Singh RV Crous PW Kukwa M Lücking R Kurtzman CP Yurkov A Haelewaters D Aptroot A Lumbsch HT Timdal E Ertz D Etayo J Phillips AJL Groenewald JZ Papizadeh M Selbmann L Dayarathne MC Weerakoon G Jones EBG Suetrong S Tian Q Castañeda-Ruiz RF Bahkali AH Pang K-L Tanaka K Qin DD Sakayaroj J Hujslová M Lombard L Shenoy BD Suija A Maharachchikumbura SSN Thambugala KM Wanasinghe DN Sharma BO Gaikwad S Pandit G Zucconi L Onofri S Egidi E Raja HA Kodsueb R Cáceres MES Pérez-Ortega S Fiuza PO Monteiro SJ Vasilyeva LN Shivas RG Prieto M Wedin M Olariaga I Lateef AA Agrawal Y Fazeli SAS Amoozegar MA Zhao GZ Pfliegler WP Sharma G Oset M Abdel-Wahab MA Takamatsu S Bensch K de Silva NI De Kesel A Karunarathna A Boonmee S Pfister DH Lu Y-Z Luo Z-L Boonyuen N Daranagama DA Senanayake IC Jayasiri SC Samarakoon MC Zeng X-Y Doilom M Quijada L Rampadarath S Heredia G Dissanayake AJ Jayawardana RS Perera RH Tang LZ Phukhamsakda C Hernández-Restrepo M Ma X Tibpromma S Gusmao LFP Weerahewa D Karunarathna SC (2017) Notes for genera: Ascomycota. Fungal Diversity 86(1): 1–594. 10.1007/s13225-017-0386-0

[B70] Zoller S Scheidegger C Sperisen C (1999) PCR primers for the amplification of mitochondrial small subunit ribosomal DNA of lichen-forming ascomycetes. Lichenologist 31(5): 511–516. 10.1006/lich.1999.0220

