# Supplementary material for: ﻿Pseudolepraria, a new leprose genus revealed in Ramalinaceae (Ascomycota, Lecanoromycetes, Lecanorales) to accommodate Leprariastephaniana
Source: MycoKeys. 2023 Mar 24;96:97–112. doi: 10.3897/mycokeys.96.98029 (PMC10210240; doi:10.3897/mycokeys.96.98029)
Supplement: Supplementary material 1 — The PCR parameters [file mycokeys-96-097-s001.docx]

Supplementary Materials

Table S1 The PCR parameters.

| Primers | ITS1F/ ITS4A | mtSSU1/mrSSU3R | ITS4A-5'/LR5 | fRPB2-5F/fRPB2-7cR | Al1500bf/ITS4M |
| --- | --- | --- | --- | --- | --- |
| Initial denaturation | 94˚C, 2 min | 94˚C, 3 min | 95˚C, 5 min | 94˚C, 2 min | 95˚C, 3 min |
| Denaturation | 94˚C, 30 s | 94˚C, 1 min | 95˚C, 1 min | 94˚C, 1 min | 95˚C, 45 s |
| Annealing | 60˚C, 1 min | 52˚C, 1 min | 60˚C, 1 min | 1. 60-56˚C (×7 decrease 0.5 ˚C), 1 min 30 s  2. 56˚C (× 33), 1 min 30 s | 51˚C, 40 s |
| Elongation | 72˚C, 1 min | 72˚C, 1 min | 72˚C, 1 min | 1. 72˚C, 1 min 45 s  2. 72˚C, 2 min | 72˚C, 1 min 20 s |
| Final extension | 72˚C, 7 min | 72˚C, 7 min | 72˚C, 7 min | 72˚C, 10 min | 72˚C, 10 min |
| Number of cycles | 35 | 35 | 35 | 7+33 | 35 |
